# A Report on the Public Health Response to Alpha-Gal Syndrome in the United States

**DOI:** 10.4269/ajtmh.26-0361

**Published:** 2026-11-03

**Authors:** Gilbert J. Kersh, Johanna S. Salzer

**Affiliations:** Rickettsial Zoonoses Branch, Division of Vector-Borne Diseases, Centers for Disease Control and Prevention, Atlanta, Georgia

## Abstract

Alpha-gal syndrome (AGS) is a unique, life-threatening tick-borne disease. It typically presents as an allergy to meat and other mammalian products because of the presence of IgE antibodies against the carbohydrate galactose-alpha-1,3-galactose (alpha-gal). Alpha-gal syndrome is induced by bites from certain tick species that naturally contain alpha-gal in their saliva. In some people, tick bites can elevate levels of alpha-gal IgE antibodies. In the United States, lone star tick (*Amblyomma americanum*) bites are the most common cause of AGS. The public health response to AGS requires education of health care providers and the public, novel methods for lone star tick control and tick bite prevention, improved surveillance for AGS, and evaluation of medical management strategies. A more specific diagnostic laboratory test would improve both diagnosis and surveillance, and tests to evaluate levels of alpha-gal present in food and other products would facilitate dose–response studies to better define the risk of reactions.

## INTRODUCTION

Alpha-gal syndrome (AGS) is a unique tick-borne disease that is a life-threatening allergy to mammalian meat and other mammalian products. It is mediated by IgE antibodies to the galactose-alpha-1,3-galactose (alpha-gal) carbohydrate, which is widely expressed on bacterial, viral, and mammalian glycoproteins and glycolipids. It is present in the tissues of nearly all mammals but absent in humans, apes, and Old World monkeys. The presence of IgE antibodies against alpha-gal resulting in allergic reactions in humans was first described in patients receiving the monoclonal antibody cetuximab for treatment of colorectal cancer in 2008.^1^ Subsequently, patients presenting with allergic reactions to mammalian meat 2 to 10 hours after consumption were shown to have pre-existing IgE antibodies reactive to alpha-gal.^2^ Further analysis of patients led to the description of AGS, where patients have delayed allergic reactions after consumption of mammalian meat or other products derived from mammals and/or more immediate reactions after exposure to medicines containing alpha-gal.^3^ Alpha-gal syndrome is considered predominantly an adult-onset allergy, and these allergic reactions develop after a long history of meat consumption without incident. Since AGS was first identified nearly two decades ago, scientific studies and public health initiatives have strengthened our understanding of its geographic and epidemiologic patterns, improved patient outcomes, and informed prevention efforts in the United States.

## ALPHA-GAL SYNDROME IS A TICK-BORNE DISEASE

Early descriptions of AGS patients and their geographic distribution led to the speculation that tick bites could cause people to be sensitized to alpha-gal.^4^ Patients allergic to mammalian meat in Australia, where this condition is called the mammalian meat allergy, were reported to have large local reactions to tick bites that preceded the onset of meat allergy by up to 6 months.^5^ Multiple studies reported that patients in the United States could recall tick bites before experiencing AGS symptoms,^6^ and alpha-gal-reactive IgE antibodies were shown to increase in three subjects after tick bites.^4^ Cases of delayed allergic reactions to mammalian meat have been reported from several countries, with evidence of linkage to a variety of tick species.^5^^,^^7^^,^^8^ A case–control study conducted in North Carolina was reported in 2023, and it showed that recollection of a tick bite was much more common in AGS patients and was a major risk factor for AGS.^9^ Tick bites were also a risk factor for the presence of alpha-gal IgE in the controls, suggesting that the role of tick bites in AGS is to induce alpha-gal IgE antibodies.^9^ Other studies have demonstrated the presence of alpha-gal in tick saliva^10^ and shown that tick saliva can induce alpha-gal IgE antibodies in alpha-gal-deficient mice when injected intradermally or when introduced through a tick bite.^11^^–^^13^ The natural role of alpha-gal in tick saliva is not entirely clear, but it has been suggested that the presence of alpha-gal on tick glycoproteins and glycolipids can facilitate tick feeding. Certain species of ticks seem to be able to produce their own alpha-gal rather than acquire it through feeding.^14^^,^^15^ Alpha-gal syndrome is, therefore, a unique noninfectious tick-borne disease, and it has the potential to be prevented by using prevention practices recommended for other tick-borne diseases.

## GEOGRAPHIC DISTRIBUTION AND ASSOCIATED TICK SPECIES

Our current knowledge of the geographic distribution of AGS in the United States is largely based on an investigation of commercial diagnostic laboratory test results from 2017 to 2022.^16^ This investigation showed the highest incidence of individuals testing positive in Arkansas, Missouri, Tennessee, Kentucky, and Virginia. These are states where the lone star tick (*Amblyomma americanum*) is the most active human-biting tick, suggesting that bites from lone star ticks are the primary cause of AGS in the United States. However, the report showed some positive test results outside the established range of the lone star tick. Alpha-gal syndrome associated with bites from blacklegged ticks (*Ixodes* spp.) has been reported from both Maine and Washington,^17^^,^^18^ indicating that lone star ticks are not solely responsible for causing AGS in the United States. Laboratory studies have demonstrated the presence of alpha-gal in saliva of *Amblyomma* spp. and *Ixodes* spp. ticks.^10^ Although bites from several species of ticks induce alpha-gal IgE, the lone star tick seems especially effective at IgE antibody induction. In some states where the lone star tick is widespread, high seroprevalence among healthy blood donors has been observed. In Arkansas, Missouri, Tennessee, Kentucky, and Virginia, the seroprevalence of alpha-gal IgE among healthy blood donors sampled in 2024 and 2025 is estimated at 24.0%.^19^ Thus, areas with established populations of lone star ticks have the majority of people with alpha-gal IgE and the majority of AGS cases in the United States. Novel methods to reduce lone star tick populations and prevent lone star ticks from biting people are needed to prevent new cases of AGS.^20^

## BURDEN OF AGS IN THE UNITED STATES

The public health response to AGS requires a clear understanding of the number and geographic distribution of people currently living with AGS in the United States. This can be done by traditional disease surveillance where state, local, and tribal health jurisdictions make AGS a reportable condition in their jurisdiction. In jurisdictions where AGS has been made reportable, public health authorities receive reports of positive alpha-gal IgE test results and conduct case investigations to classify cases. The Council of State and Territorial Epidemiologists approved a standardized case definition that has been active since 2022 to support uniform classification across jurisdictions. Confirmed cases of AGS require a positive alpha-gal IgE test result and compatible clinical criteria, which include allergic responses that commence 2 to 10 hours after ingestion of alpha-gal-containing products or within 2 hours after intramuscular, intravenous, or subcutaneous administration of an alpha-gal-containing vaccination or medication. Determining if the case meets the clinical criteria typically requires evaluation of medical records and/or communication with the health care provider or patient. For states in areas where people are often bitten by lone star ticks, this can mean thousands of case investigations per year (CDC, unpublished data). The effort needed to conduct case-based surveillance for AGS exceeds the capacity of states to conduct investigations. Because AGS has not been made nationally notifiable by the Council of State and Territorial Epidemiologists, states are not under obligation to report the surveillance numbers to the CDC. Although traditional surveillance for AGS is still in the early stages and does not yet provide comprehensive data for the number of cases in the United States, it is clear that for some states, AGS is the most common vector-borne disease.^21^

An alternative method to provide an estimate of AGS prevalence is to examine alpha-gal IgE clinical laboratory test results. From 2010 to 2022, alpha-gal IgE testing in the United States was done primarily by a single commercial laboratory. In that time, more than 110,000 persons received a positive alpha-gal IgE test result from this laboratory.^16^^,^^22^ Assuming that at that time, 80% to 90% of these persons with positive alpha-gal IgE test results also had compatible clinical symptoms^16^ and that most people with AGS do not have access to a health care provider with enough knowledge to order the alpha-gal IgE test,^23^ it was estimated that as many as 450,000 people had AGS during 2010–2022. In the years since then, several major commercial laboratories have started offering alpha-gal IgE testing, and tests have been ordered more frequently. The rise in testing may be related to an increase in cases of AGS, a growing awareness of AGS, or testing of patients who do not have symptoms of AGS but were nevertheless tested because of a history of a tick bite or other noncompatible clinical reasons.

A major issue in reliance on laboratory testing data alone to estimate the number of cases of AGS is the high prevalence of alpha-gal IgE antibodies among people living in areas where lone star ticks are common. Seroprevalence of alpha-gal IgE in people who do not have AGS can be more than 30%,^9^^,^^19^ and if there is increased testing of patients without the requisite symptoms, the value of the alpha-gal IgE test for surveillance diminishes. The public health investigations required to evaluate clinical symptoms alongside IgE testing data could strain the resources of the most impacted states. A more specific laboratory test for AGS would greatly facilitate surveillance for AGS if it could lessen the need for case investigations. Despite these limitations, laboratory testing data suggest that the trajectory of AGS cases in the United States is following that of other tick-borne diseases^16^^,^^22^ and that it is expected to continue to rise as lone star tick bites become more common^24^ and knowledge of AGS grows.

## PATIENT MANAGEMENT AND PROGNOSIS

The primary method for clinical management of AGS is to eliminate alpha-gal from the diet. The level of dietary restrictions needed varies among patients depending on individual sensitivity to alpha-gal. Many patients report tolerating dairy products and even small portions of mammalian meat, but others are unable to even tolerate fumes from meat being cooked nearby.^25^ The severity of reactions can also vary, with some reporting only mild symptoms, but other reactions are severe enough to cause death.^26^^,^^27^ An understanding of why some patients have more severe reactions and the contributions of cofactors, such as exercise and alcohol consumption, are important for patient management.^28^ Removal of all alpha-gal from a patient’s diet can be difficult as mammalian products are often added to foods that would not be considered meat or dairy by most consumers.^29^ Medicines, makeup, and lotions can also contain mammalian ingredients and represent potential exposure. Classifying alpha-gal as a major food allergen has been suggested as a way to help patients as this would require labeling of foods that contain alpha-gal. The usefulness of food labeling would be improved by having a standard method to measure alpha-gal in food products and having dose–response data to determine sensitivity across the AGS patient population. The difficulties in maintaining a strict avoidance diet means that accidental exposures are common for AGS patients. Oral antihistamines, oral corticosteroids, epinephrine autoinjectors, and the anti-IgE antibody omalizumab have all been suggested for use in medical management of AGS.^29^ Clinical trials to test the efficacy of these treatments and other medical management strategies are needed. Tick bite prevention is a key component of AGS management^29^^,^^30^ and may allow patients to eventually experience a reduction in alpha-gal IgE titers and allergic reactions, with the timeline for clinical improvement varying widely among patients.^29^^,^^31^

Nonallergic people with alpha-gal IgE antibodies are said to be “sensitized,” and high rates of sensitization have been reported in several populations.^9^^,^^19^^,^^32^ There is currently no evidence to support dietary modifications in sensitized people,^33^ but allergic transfusion reactions have been reported in sensitized patients.^34^^,^^35^ Additionally, sensitization has been linked to obstructive coronary artery disease^36^ and may have some impact on accelerated bioprosthetic heart valve degeneration.^37^ The potential health implications of sensitization should be further studied to allow for better risk stratification.

## PUBLIC HEALTH RESPONSE TO AGS

Although knowledge about AGS has increased greatly since its sudden emergence in 2008, there are still large deficiencies in AGS knowledge among the public and health care providers.^23^ Many patients have reported requiring more than 7 years to receive a diagnosis of AGS,^38^ with recent evidence suggesting that this timeline may be shortening.^39^ Education of both health care providers and the public will continue to be a key component of the public health response to AGS. In the fall of 2025, the CDC released an online health care provider training module to address these gaps in provider knowledge of AGS and shorten the time to diagnoses for patient across the United States.^40^

Future components of an AGS public health response will be facilitated by technological advances. Food labeling will benefit patients but is complicated by lack of quantitative tests to detect the alpha-gal carbohydrate and dose–response analysis in patients. Surveillance for AGS is difficult because of the high background seroprevalence of alpha-gal IgE and the resulting requirement for a detailed clinical history for each patient. A more specific laboratory test is needed. Finally, prevention of AGS will require novel methods of lone star tick control and bite prevention, emphasizing the need for involvement of entomologists in this public health effort.^41^
